# Viral Law: Life, Death, Difference, and Indifference from the Spanish Flu to Covid-19

**DOI:** 10.1007/s11196-022-09893-7

**Published:** 2022-03-18

**Authors:** Mark Featherstone

**Affiliations:** Sociology, Keele University, Staffordshire, ST55BG UK

**Keywords:** Virus, Freud, Lacan, Derrida, (In)Difference

## Abstract

What is viral law? In order to being my discussion, I note that the last two years have been extremely difficult to understand and that we, meaning those who have lived through the pandemic, have struggled to make sense. Thus, I make the argument that the virus has impacted upon not only the individual’s ability to make sense in a world where every day routines have been upended, but also social and political structures that similarly rely on repetition to continue to function. According to this thesis, Covid-19 is more than simply a biological organism, but also a cultural virus that undermines the organisation of social, political, and economic systems and requires new ways of thinking about how we might move forward into a post-Covid world. In the name of beginning this project of making sense of Covid-19, I track back in history to the comparable reference point of the Spanish flu pandemic of 1918–1920 and, in particular, a reading of Freud’s *Beyond the Pleasure Principle*, which the founder of psychoanalysis wrote in the shadow of the virus. In reading Freud’s attempt to write a psychology of death in the context of this funereal period of history, I argue that he set out first, a mythological theory of viral law concerned with the death drive, before turning to second, a techno-scientific, biological theory of the same (viral) law characterised by microbial immortality. Beyond this exploration of *Beyond the Pleasure Principle*, in the third part of the article I turn to a reading of Lacan’s interpretation of Freud’s work, where viral law becomes a story of cybernetics and nihilistic mechanisation. Here, perfect mechanisation, and the endless oscillation between message and noise, looks a lot like living death. Finally, I take up Derrida’s critique of Jacob’s molecular biology and, by extension, Freud’s theory of microbial immorality, that he thinks privileges an idea of repetitive sameness and opens up a space for cultural politics concerned with immunity against otherness. Derrida’s key point here is that this biological fantasy ignores the reality of viral sex that enables evolution to happen. What this means is that the other, even in its microbial form, is ever present, and that we must recognise the importance of difference to the possibility of social, political, and economic change.

## Making Sense in Dark Times

Looking back from the perspective of January 2022 it is uniquely difficult to make sense of the events of the last two years. We, meaning those who have lived through the pandemic, have been confused, disorientated, and perhaps even traumatised in subtle ways that mean that we can no longer make sense. We are desperately in need of what Priscilla Wald [1] calls an outbreak narrative that would allow us to understand what has happened. In her book *Contagious* [1] Wald traces the history of the outbreak narrative from typhoid to Aids and shows how societies make sense of epidemics and pandemics that ravage their populations. There have, of course, been versions of this kind of sense making in the current context. These have taken the form of brazen politically motivated stories surrounding the ‘China Virus’ and ‘Kung Flu’ or outlandish global conspiracies concerning the influence of the illuminati. Although these narratives might work on the fringe they would seem to offer little from a serious point of view. Having said this, we must recognise that the history of thinking through epidemics and pandemics has been framed by racist, colonialist, sexist, and homophobic understandings, as Wald illustrates through her discussions of Typhoid Mary and Gaetan Dugas (Patient Zero in the Aids pandemic). However, there are severe political limitations to these kind of narratives. They may appear to immunise against the contagious nature of the other, but there are serious problems with this approach when we recognise that relationality is irreducible, a fact of life, particularly in a globalised society. We have to live together. The alternative to long term, once and for all immunity, is that we learn to live with the other in its viral, animal, and human form and recognise our own finitude. It may be, then, that this is the essential question for a politics of the post-Covid period. How can we learn to live with the other in a world where immunity would *seem* like the best option?

This is precisely what the philosopher of illness Havi Carel [2] explores in her work. Explaining that long term illness brings about a loss of bodily certainty, wholeness, and the ability to make sense, Carel argues that the sick person who experiences the transformation of their body into an object that resists their will, must learn to accept their own finitude. The sick person very quickly learns that they are not a god, that they are not in complete control, and that they will not live forever. Since the body is the medium through which we relate to the world, Carel explains that corporeal doubt, and the sense that the body is no longer reliable, means that our world is also thrown into doubt and begins to disappear before our eyes. Suffering, struggling to manage their sickness, the sick person cannot imagine a future beyond their current condition, and it is easy for them to slide towards depression. However, Carel notes that in these circumstances it is important that the sick person does not fall into a state of thanatophobia, where they become crippled by a fear of mortality, and seek to compensate for this through the denial of finitude. Instead, she suggests a focus on finitude in the manner Heidegger [3] writes about in the context of his theory of being-towards-death because this will allow the sick person to begin to make sense of their life and what is possible in their situation. I think that this is precisely the challenge we face in the wake of the Covid-19 pandemic, which swept away the bodily certainty of those who became most severely ill with the virus, but has also seriously damaged the more general social, political, economic, and, perhaps most importantly, cultural systems that suture individuals into the world on a psychological level. Although society may have staggered on through the last two years, the wheels of the economy have continued to turn, and political leaders have managed to lurch from one disaster to another, our cultural systems, the structures that provide the psychological link between the individual and the group, have started to come apart at the seams, with significant impacts upon individuals who live with reference to socio-symbolic law.

Moreover, this is not simply a problem for those struggling to breathe, or those suffering the debilitating effects of long Covid, who have seen the world disappear from view from the confines of their ‘sick rooms’, but also everybody else who has found themselves ‘locked down’, confined to home, and thrown back upon their own individual psychological reserves. In much the same way that we make sense together, developing a cultural world that enables us to understand events, we also endure in common, surviving the cruelties and turbulence of the world by supporting each other on an everyday basis. Basic routines shared in common enable us to maintain a sense that the world continues to turn and help us to understand the relationship between past, present, and future in order to construct meaningful lives. Although the phenomenologists who developed this theory of the ways in which we live in time, the existential tendencies of Heidegger and Sartre meant that they had little time for what they would have considered inauthentic or serial forms of temporality. However, under conditions of Covid lockdown even the most basic forms of everyday routine, which normally enable people to understand the link between past, present, and the future have collapsed to be replaced by a kind of grey monotony framed by the four walls of their homes turned carceral space. Caught in a situation resembling the scenario Sartre [4] sets up in his *No Exit*, Hell is simultaneously the other people one cannot escape, but also the lack of other people similarly confined. Hell is also endless monotony, the law of the same that produces indifference, boredom, and eventually the looming anxiety that emerges when one realises that the everyday life of banal routine that had kept the world turning no longer really exists. Under these conditions, even the medium-term future disappears from view. Thus, the future becomes a kind of black hole and the existential challenge becomes to shine a light into the void and begin to make sense. In Heideggerian and Sartrean terms, the challenge is for us to develop a project in order to move beyond what we might call ‘the viral end of history’.

While this struggle for identity is one which everybody must face, we know that the post-pandemic future is not simply about the existential problems of the lonely individual. The precise reason I refer to the idea of ‘the viral end of history’ above is because it seems to me that the last two years might be best understood in terms of the definitive end of the end of history period first announced by Frances Fukuyama [5] in the late 1980s/early 1990s. Following Fukuyama’s claim that the end of the Cold War marked the end of serious ideological change on a global level and that the future would be American, the American empire soon began to crumble. After 9/11, and the emergence of the virus of fear, terror, and insecurity, in 2008 contagion spread through the financial markets throwing the global economic system into doubt and leading many to question the future of capitalism itself. The Covid-19 pandemic has only further undermined the authority of the American-led global system. According to the WHO (World Health Organisation) America has the highest reported Covid death toll, as I write almost 1,000,000 souls, which is more American lives lost than in World War I, World War II, and Vietnam combined. Given the incompetence of Trump, who spent much of the first wave of the pandemic trying to deny the severity of the virus before shifting to a blame narrative focused on the Chinese origins of the infection, and the decrepitude of Biden, who appears to be the perfect embodiment of a nation suffering in a state of old age and ill health, it is no wonder that the cultural systems that have sustained the west on a social, political, economic, and individual psychological level since at least the end of World War II feel broken and in need of rethinking. The question we must ask then is what comes after the American century? How can we move beyond the Covid-19 pandemic? How can we understand the political stakes of the viral infection that has wreaked havoc over the course of the last two years and make sense of our possible futures?

Now, in what follows I propose to construct a cultural politics of the Covid-19 pandemic in the name of opening up a debate about how we might move beyond the crisis into the future. In order to develop this cultural theory, I propose to track back in history to the last comparable reference point for making sense of Covid-19, the Spanish flu pandemic of 1918–1920. From this point, I move forward in history, offering a microbial, molecular, bio-political history of the 20th century moving into the 21st century. Following a discussion of the immediate post-World War I years marked by the Spanish flu, I consider the post-World War II period characterised by the emergence of molecular biology, the discovery of the DNA program, and what we might call viral sex. In the context of exploring this historical period, and in the name of setting out a cultural political theory of Covid-19 concerned with how we might think about moving beyond the pandemic, I base my discussion upon a number of key texts. As my discussion evolves these texts come together to form a coherent debate about the meaning of what I am calling ‘viral law’, which concerns thinking about the way the virus and viral society might move into the future on the basis of a law-like regulatory system.

Starting with the Spanish flu pandemic that raged across Europe and rest of the world between 1918 and 1920, and which Laura Spinney writes about in terms of a kind of void of immediate sense and historical memory, I read Freud’s [6] classic text, *Beyond the Pleasure Principle* in order to develop an initial theory of the meaning of viral law. My objective here is to set out a theory concerned with what Derrida [7] calls Freud’s auto-thanato-biography. That is to say that my argument is that Freud’s *Beyond the Pleasure Principle* is overwhelmingly coloured by his own feelings about death and the pervasive atmosphere of death, decay, and decrepitude that marked the period in which he was writing. My thesis is that in the face of this funereal atmosphere characterised by World War I and the death of his daughter Sophie, Freud ended up imagining the possibility of a completely immune ego, free from the horrors of finitude, in the form of a microbe, the famous protozoan discussed in chapter six of the text, which lives forever through the process of endless repetition and cellular division. Although *Beyond the Pleasure Principle* [6] is, in many ways, Freud’s most focused exploration of death, there is another sense, supported by Razinsky [8], in which Freud could never truly confront mortality, and instead skirted around the issue that one day there will be no more days. Arguing that Freud turns to the work of August Weismann and the theory of the immortal germ cell to defend against the spectre of death that seemed everywhere from 1914 onwards, my claim is, therefore, that the father of psychoanalysis developed an idea of viral law, the law of the endlessness of germinal life, in a social and cultural context where mourning was impossible because of the sheer scale of the human catastrophe taking place. Disorientated by the invisible nature of the influenza virus, my point is that it became impossible to lay the victims of the flu to rest, with the result that a generation already traumatised by the horrors of World War I suffered from what Laurence Rickels [9] calls aberrant mourning or a fear of mortality represented by feeling haunted by spectres, ghosts, and the spirits of those who have passed, but not moved on because their deaths have not been effectively mourned. In my reading, *Beyond the Pleasure Principle* [6] is marked by this logic, the logic of what Rickels calls ‘unmourning’, and that this is why Freud speculates that the protozoan represents the possibility of the abolition of death. In short, Freud seeks to perform a textual exorcism.

Building upon my discussion of Freud’s *Beyond the Pleasure Principle* [6], in part three of my article I move on to explore Derrida’s [7] critique of Francois Jacob’s molecular biological theory of DNA and programmatic life in the context of the post-World War II leap beyond Nazism, authoritarianism, and the long history of fantasy of eternal utopia that runs from Plato through More up to Marx. The contextual, historical, point here is, of course, that in much the same way that Freud sought to evade the horror of death caused by World War I and the virus that seemed to spread with unstoppable force by imagining an alternative kind of viral law of the simple singular celled organism that lives forever by endlessly dividing itself, the Nazis would go on to try to immunise the Germans against all others, but particularly the Jews who they thought would infect the pure blood of the Aryan race if they were not exterminated. While Freud would later write, in *Civilization and its Discontents* [10], that we have to put up with the misery of civilization and learn to live with others, because the alternative to this, the pursuit of the utopian Nirvana complex, would result in death and destruction on a global scale, the Nazis followed the death drive to the very end in Hitler’s Berlin bunker on 30^th^ April, 1945. It is in this context that I argue that Derrida sought to deconstruct Jacob’s similarly closed system of molecular biological programmatic life on the basis that what this theory really set out was an update on Weismann’s vision of the germ cell that lives forever, which Freud had adopted in his psychological defence against the funereal atmosphere caused by the Spanish flu pandemic. This is precisely why Derrida [7] shifts from his critique of Jacob’s system, which presents a theory of the true form of the living in terms of unicellular organism that has no need for otherness, to a discussion of Freud’s auto-thanato-biography and his adoption of Weismann’s theory of the germ cell that lives on as an alternative to the death drive concerned to end it all as soon as possible. In Derrida’s account, the problem with this logic, which runs through Jacob’s work and before this Freud’s auto-thanato-biographical turn to Weismann, is that the denial of the other, in its molecular, microbial, and more complex human form, is that it is founded upon a fantasy, a utopian model of life, that does not exist, principally because the phenomenon of microbial, viral sex means that the solipsistic cell/egoistic self is always in communication with others, regardless of the *theoretical* possibility of total immunity against the outside. In this respect, the Freudian vision of somehow living beyond death, the Nazi fantasy of a Germanic utopia free from others, and Jacob’s molecular biological program that simply repeats ad nauseam, are impossible utopias that ignore the irreducible reality of communication, sex, and as a result death and finitude.

## Freud and the Unicellular Utopia

In order to begin my cultural political history of modern understandings of viral infection and pandemics, in this section of my article I return to the case of the Spanish flu of 1918–1920 and take up Freud’s *Beyond the Pleasure Principle* [6], which was written in the period immediately following World War I and in the middle of the influenza pandemic that would claim the life of Freud’s favourite child, his daughter Sophie. As explained above, my basic thesis is that surrounded by death and the deadly contagion of the flu, Freud imagines Thanatos, the death drive that suggests that the human’s first instinct is to return to a state of inanimate matter and nothingness in order to escape from the traumas of life. According to Freud, this is hard-wired into humans through what he calls the inertia of nature, which confirms that the tendency towards death is not simply a human psychological phenomenon, but rather a biological fact of the natural world. Although there is life, death is ever present, and all things must die in the end. In the context of setting out this thanatological law, we might conclude, then, that Freud’s first version of viral law is the death drive itself, which we could speculate is a representation of what he had lived through since 1914. Indeed, this speculative thesis would be, to some extent, strengthened through a consideration of *Civilization and its Discontents* [10], where Freud lifts his analysis of the death drive towards a sociological level and considers the relationship between a will to self-destruction, which is then projected into aggression, that finds form in the history of warfare. While this reference connects the death drive to the events of 1914–1918, it is harder to establish a clear textual link to the virus, but I would argue that the relationship between the reality of the influenza pandemic and the idea of the death drive resides in the sense in which the viral organism seemed to possess a kind of demonic force that drove its contagious nature. As far as I am aware Freud never comments on this connection between the imaginary of the virus and the demonic drive, but I believe that this shadow version of viral law represents a kind of hidden, or perhaps even unconscious, pretext to the famous chapter on the protozoan (chapter six) where Freud imagines an alternative to the endless death drive, Thanatos. In this case, Weismann’s biology, the idea of the germ cell, and the protozoan that lives forever without the inconvenience of death represents the biological support for the endless life drive, Eros. Given this reading of *Beyond the Pleasure Principle* [6], my thesis is that Freud conjures the idea of the endless life drive of the microbial organism to try to manage his own sense of doom and gloom in the face of the great war and Sophie’s demise in the pandemic. This is, then, my theory of Freud’s viral law: first, the experience of the Spanish flu pandemic, which Freud imagines through the myth of the law of the demonic death drive that compels the living to return to a state of nothingness and non-existence, before he turns to second, the alternative counter-law of the immortal microbial life, the germ cell, the sexless protozoan that divides endlessly without passing through the horror of death.

Writing on the relationship between Freudian psychoanalysis and the current pandemic, Brett Kahr [11] supports this view of the psychological backstory to *Beyond the Pleasure Principle* [6] by painting a picture of Freud constantly reminded of his own finitude and mortality. From World War I, through the Spanish flu pandemic and the loss of his daughter, into the 1920s and 1930s when Freud struggled with continual pain from his cancer of the jaw and the ever-present threat of the Nazis, Kahr shows how Freud’s life was characterised by a constant struggle to survive in the face of the spectre of death. On the basis of this insight, the key point of Kahr’s [11] book, and his rationale for reading Freud in the time of Covid-19, is to think through what Freud might have made of the current pandemic on the basis of the ways in which he handled his own problems in the early part of the 20th century. Kahr's question is, then, about how we might make sense of Covid-19 with reference to how Freud made sense of the Spanish flu pandemic a century ago. According to literary theorist Elizabeth Outka [12], this question of the significance and meaning of life in the face of the spectre of death remains central to understanding the history of the Spanish flu. In much the same way that we struggle to make sense of Covid-19 today, Outka’s outstanding book, *Viral Modernism* [12], which was published in the months immediately prior to the outbreak of the Covid pandemic, is really about how modernist writers, such as Virginia Woolf and W. B. Yeats, sought to capture the experience of the Spanish flu through the modernist literary form. Reflecting the idea that the deaths caused by the flu where somehow less grievable than those resulting from World War I, simply because it was possible to explain the events of the war in terms of rational cause and effect relationships, human decision-making, and the span of historical events, Outka makes the key point that the flu was resistant to traditional narrative structure, meaning that it was a kind of perfect modernist event—nauseating, disorientating, and seemingly unknowable.

Before the invention of the electron microscope in the 1930s, it was, of course, impossible to observe the virus, meaning that it took on a kind of spectral, supernatural quality, that Woolf and the other modernists sought to represent through their own strange, delirious, texts. Unlike the contemporary Covid-19 period, where molecular biologists were able to observe, know, and sequence the virus more or less immediately, and populations could learn about and experience the contagious organism through the mediasphere, the invisibility, unknowability, and unpredictability of the Spanish flu virus meant that it was not possible to easily explain what was happening through normal narrative form. Instead, Outka shows how the modernists sought to reflect the social, psychological, and cultural impacts of the virus in the very ways in which they sought to make sense. Thus Woolf, Eliot, and Yeats would try to capture the way the virus corrupted the individual and destroyed previously reliable social structures through texts marked by an internal, formal sense of fragmentation, delirium, disorder, and a spectral atmosphere full of ghostly hauntings. As Outka [12] suggests, the virus itself seemed to be possessed by a kind of drive, endlessly circulating the new landscape of death, with the result that those already suspicious of the other now had good reason to suspect their neighbours might be harbouring the deadly infection. In the case of those struck down by the virus, caught somewhere between life and death much like the enigmatic organism itself which biologists still talk about in terms of its zombie-like properties, Outka says that it was impossible to make sense. The everyday narrative form, which the phenomenologists would show enable us to connect to the world, collapsed before the pain and suffering that unmakes language. In his classic poem written in the teeth of the pandemic, *The Second Coming* [13], Yeats imagines the apocalypse—‘Things fall apart, the centre cannot hold; Mere anarchy is loosed upon the world’—and in this way seeks salvation in religious thought. When there is no hope, we must turn to God. Despite Nietzsche [14], who announced that God has left the stage at the end of the 19th century, the Spanish flu seemed to make Him entirely necessary. In the face of the new contagion modernity and the human-made world was found wanting.

My key point is that I think that we might understand Freud’s *Beyond the Pleasure Principle* [6] through this lens. That is to say that under conditions where the virus remains impossible to observe, identify, or know, it is possible that the scientist within Freud turned back to mythology, and the figure of Thanatos to imagine an unstoppable, demonic force within nature set upon the compulsion of the living to return to an inanimate state. Although Freud was clearly aware that the flu was the result of infection by some external contagious organism, and the point remains that he imagined the death drive as an internal force, it is entirely possible that the invisibility, unknowability, senselessness, and apparently unstoppable nature of the virus inspired Freud to imagine that humanity was indeed possessed by a drive to escape from the pain of life into the peace of nothingness. Better to sleep, and disappear into the darkness, than to live with constant pain and suffering. Should we take this view, and imagine that Freud’s experience of living through the Spanish flu pandemic provided inspiration for the concept of the death drive, then we might conclude that his *first* viral law was the thanatological law concerned with the drive towards death and (self-)destruction. The reason I emphasise the word ‘first’ in this context is because we can see that Freud’s next step is to shift his focus back from the mythological figure of Thanatos to science and August Weismann’s microbial protozoan that lives forever through repetition, replication, and cell division in order to neutralise the mythic viral drive to extinguish life. In this *second* viral law, which takes the form of the simple, single celled organism, there is no contagion because there is no other, no sexual intercourse, and no space for infection.

Thus, we might conclude that Freud [6] imagines a viral law of immortal life in opposition to his viral law of endless death in such a way that provides some respite from the nightmarish conditions of the present and mythological support for the idea of the ego that he believes needs to stand on its own and resist the forces of unconscious contagion. We know that Freud thought that this was essential. The human must make the leap from the contagious universe of nature, represented by the initial connection between mother and child, towards the world of splendid isolation characterised by Oedipus and the father, where loss, loneliness, and trauma become normal states that the newly socialised child must learn to endure in the name of civilization. That Sophocles’ Oedipus Rex only really became Freud’s Oedipus of the psychoanalytic complex and defender of the incest taboo after surviving the plague of Thebes and coming to know himself and his crimes, further supports the conclusion that Freud imagined that civilization was located on the other side of the thanatological viral law of destruction, nothingness, and mythological thought in a second erotic viral law of life, order, and, perhaps most centrally, rational knowledge and scientific understanding. The key transformer or shifter between the two forms of viral law is, therefore, one that comprises knowledge, reason, and scientific knowledge. What really matters in this context then is the ability to shed light on the virus, to move it from a primitive mythological to modern scientific space, and to make sense. This is, of course, also the basic shift required for the foundation of civilization, which is most clearly expressed in essays such as *Repression* [15] and *Civilization and its Discontents* [10], where Freud explains that the primitive in the human must be controlled (repressed) to ensure their survival. Conversely, however, this move to Oedipus, repression, and civilization is precisely what conjures the obsession with a return to a pre-Oedipal state, a more basic Darwinian world of escape, contagion, and continuity, a world, in other words, where the *first* viral law, the law of death and destruction, rules over the behaviour of the human-animal.

We recall that this is precisely how Freud [6] opens up his debate about the death drive in the first place. Everything starts from Freud’s observations of his grandson, Ernst, son of Sophie who will soon die of the flu, playing the simple game Freud will later call fort/da. The idea is that in order to cope with the trauma of his mother leaving the room, Ernst throws his toy away only to pull it back towards him, with the objective of mastering his feelings of loss on a symbolic level. Since the game cannot change the reality that mother has left the room, Freud notes that Ernst must repeat endlessly, until Sophie returns. Thus, concluding that it is possible to identify the compulsion to repeat on the other side of the pleasure principle, which is all about overcoming feelings of displeasure, discomfort, and anxiety, Freud takes off into mythological and biological speculation. First, the compulsion to repeat represents the inertia of nature, and the conclusion that everything must die in the name of the generation of new life, before Freud speculates upon the possibility of Thanatos, the death drive, and the human’s natural suicidal tendencies to seek out a return to nothingness from the moment they are born into the world and socialised into the life of a lonely ego. The next point here is key. Of course, Freud thought that the child must endure this loneliness and that mother leaving was a kind of dress rehearsal for the Oedipal upheaval to come. In the case of Ernst, this separation from mother was enforced by Sophie’s untimely death, leaving the child to live out the rest of his life in a state of loss. We know that on a conscious level at least Freud thought that this was the essence of life, and that we must come to terms with loss, which is why he was fearful of the natural tendency to escape into mortality, the death drive. Although the sociological implications of the possibility of Thanatos are not raised in *Beyond the Pleasure Principle* [6], and Freud does not speculate upon them until *Civilization and its Discontents* [10] when he introduces the notion of the Nirvana complex, it is hard to imagine that his theories of the primitive, mythological death drive, were not in some way influenced by the enormous world-historical events that he was living through in the early part of the 20th century, namely World War I and the Spanish flu pandemic.

In the face of his fear of nature, and his fear of the death drive which we suspect he might have imagined was embodied by the unknowable virus and the cold mechanisation of modern warfare, it is perhaps peculiar that Freud ends up tracking back through his own evolutionary story, comprising *Totem and Taboo* [16] with its pre-individual horde, until he reaches Weismann’s microbial God, which, in a sense, neutralises the thanatological power of the death drive, nature, and his initial mythological conceptualisation of viral law and establishes the supremacy of life, civilization, scientific thought, and the possibility of the self-identical ego produced by the Oedipal family. Despite his avowed scientism, then, I want to make the argument that Freud turns back towards mythology, and the kind of primitive thought he most surely wanted to escape from, when faced with a cultural atmosphere pervaded by death, destruction, and unmourning, through the way in which he identified humanity with Weismann’s scientific God, the protozoan that needs no others in order to live forever. Akin to Hitler, who was similarly a product of World War I and the chaos that followed, and who imagined and then sought to realise a thousand-year Reich totally immune to the contagion of the infectious Jew to ease the terrors that plagued him throughout his life, I want to make the case that an inability to mourn in a context defined by loss on a vast scale led Freud to seek escape into the immorality of the microbial God theorised by Weismann.

In responding to his own question about Freud’s survival techniques, Kahr [11] evokes not simply the talking cure, though he does point out that we need to share trauma in the name of making it bearable, but also the writing cure, because writing can enable us to essay problems and to make sense for ourselves and unknown others who may read our words. Perhaps this is how we must understand *Beyond the Pleasure Principle* [6]. Perhaps Freud essayed the pervasive atmosphere of death in the name of making sense and arriving at a vision of unicellular utopia of endless life, a kind of theologico-scientific model that could support his own civilizational idealism of family structure and egoistic independence. The paradox of this conclusion is that it was this very egoistic independence, which poor Ernst had to develop in the wake of his mother’s death, that Freud wrote both in favour of, through his evocation of the sexless unicellular utopia, and in contradiction to, through the way in which he leant on a theologico-scientific model of the splendid isolation of the microbe, in order to support his belief in the necessary independence of the ego. All of this because he himself found it impossible to live a purely scientific life and withstand the horrors of the funereal environment he inhabited at the time of writing.

## Lacan, Derrida, and the (In)Difference of Viral Law

In the previous part of this article I presented a discussion of what I am calling viral law centred upon (a) Freud’s possible representation of the Spanish flu through the notion of the death drive, which potentially mirrored the experience of living in a society wracked by the virus in respect of the way in which it represented a demonic force relentlessly pushing forward towards death, and (b) his attempt to counter this funereal vision of life on the way to death through the development of a second viral law based upon reference to August Weismann’s biology and particularly the figures of the germ cell and protozoan that reproduce endlessly without the need for otherness, sex, or death. My argument here was that ultimately Freud sought to immunise himself against the atmosphere of death circulating from 1914 through to 1920 and the publication of *Beyond the Pleasure Principle* [6] and that the creation of a kind of unicellular utopia that might provide some kind of hope for his idea of a robust, rugged, egoistic individual able to live in a civilized world and cope with loss, trauma, and finitude in the knowledge that the most basic form of life is irrepressible, unstoppable, and immortal and that it is only when we move towards more complex, multicellular organisms that the dystopia of death and the death drive enters the picture. In what follows I propose to develop this reading of Freud by moving forward into the immediate post-World War II period. Here, I move beyond Freud’s attempt to immunise against the viral law of the death drive that endlessly repeats into the future, and beyond the Nazi fantasy of a self-identical utopia free from otherness, into what has been called the American century and reference to first, Lacan’s [17] cybernetic reading of *Beyond the Pleasure Principle* [6] in his seminar on the ego and technique in psychoanalysis from the mid-1950s and second, Derrida’s [7] seminar on life and death from the mid-1970s which contains a reading in Freud’s text in the context of Francois Jacob’s classic foundational text of molecular biology, *The Logic of Life* [18].

As a result, the point of this section of the article is to project the Freudian theory of viral law set out in the second part of the piece into a reading of structuralist and post-structuralist thought concerned with the emergence of cybernetics and information theory, post-industrial high-tech capitalism, and the new field of molecular biology, which taken together would later lead to the emergence of neoliberal bio-capitalism Melinda Cooper explores in her book, *Life as Surplus* [19]. Given this background, the point of reading Lacan’s seminar is to show how he was able to translate Freud’s theory of the viral law of the death drive into a theory of cybernetics, information transfer,and repetition through feedback, thus moving the concept of viral law towards computation and suggesting the possibility of what would become the computer virus that infects the information system and then reproduces endlessly in such a way that corrupts the normal operation of the program. While this cybernetic version of the death drive appears to reflect Freud’s first version of viral law, in respect that endless repetitious viral reproduction ends up destroying the information system, there is also a sense in which Lacan’s theory suggests a kind of perfect cybernetic system, in the sense that the relentless repetitious transfers of information (communication) and feedback loops (control) present the image of an entirely rational machine. In this respect, we might make the case that Lacan’s work lifts Freud’s second theory of the immortality of viral life towards the level of the cybernetic machine and connects this back to Jacob who, in the *Logic of Life* [18], brings cybernetics, information theory, and principles of mechanisation into conversation with biology in his study of molecular life. Despite this reading of Lacan’s possible relationship to the idea of viral law, which connects his interpretation of *Beyond the Pleasure Principle* [6] to cybernetics and the notion of a perfectly functional machine, in her book *The Freudian Robot* [20] Lydia Liu suggests an alternative interpretation of perfect functionality that draws out its thanatological properties through reference to Claude Shannon’s suicide machine that performs one function: the moment the user switches the machine on, the machine switches itself back off and so on ad infinitum. What this means is that Lacan’s [17] reading of Freud’s work in the context of cybernetics essentially fuses the two versions of viral law set out in part two of my discussion by showing how the notion of an endless repetitious drive towards death (the death drive and the Spanish flu virus) and the relentless repetitious reproduction of life (Weismann’s germ cell, the protozoan, and Lacan’s perfect machine) basically collapse towards one self-identical version of the same law characterised by the kind of cancerous repetitious reproduction of the same that screens out any form of externality or otherness. This is, then, precisely where Derrida comes into the picture and how he ends up critically reading Jacob’s molecular programmatic update of Weismann’s biology and beyond Freud’s viral law of the single-celled utopia.

However, before we reach Derrida’s critique of molecular biology and Freud’s viral law, I propose to turn towards Lacan’s [17] reading of *Beyond the Pleasure Principle* [6] and the death drive as a cybernetic switching system endlessly moving between being and nothingness, life and death, and to try to position this interpretation within the social and political context of the American century and rise of computational capitalism. This contextual point is important for understanding the cultural politics of the figure of the virus. Although one might imagine that the historical shift from Europe, and the utopian tradition of thinking that eventually led to the Nazi drive for complete immunity against all others, to America, which took place in the wake of World War II, might result in the emergence of a more liberal, democratic, tolerant approach to otherness, Wald explains that America, and to an extent the rest of the western world in the American sphere of influence, was gripped by the fear of the communist virus that had the potential to produce mindless conformity and a tendency towards the endless repetition of the Party line in the masses who were uniquely susceptible to this kind of persuasion or brainwashing through the contagious nature of media, information, and other forms of viral communication. The irony was, of course, and this would not be lost on anybody who knows the history of this period, is that this virus of fear, suspicion, and creeping paranoia led to the emergence of an authoritarian brand of Americanism led by McCarthy and HUAC that seemed more like Stalinism than anything we would normally associate with American liberal, democratic values relating to freedom of speech, freedom of expression, and so on.

We know that this American tendency to seek immunity and screen out otherness in the name of protecting some sense of essential identity was subject to critical scrutiny long before Derrida’s critique of Jacob’s techno-scientific, cybernetic vision of the living that sought to marginalise relationality. Referring to science fiction films, such as *The Invasion of the Body Snatchers*, and we might add the original version of *The Thing* later remade for the bio-capital age by John Carpenter, Wald settles on William Burroughs as *the* key critic of the post-war American virus of conformity and control, citing works such as *The Ticket that Exploded* [21] and the notion of the word virus to support the thesis that the problem of viral infection and contagion was not *simply* a biological problem, but also an issue that impacted upon the social, political, economic, and cultural systems that were essentially concerned with the circulation of information. In the wake of the post-war cybernetic revolution and Watson and Crick’s discovery of DNA in 1953, this was, of course, the essential structuralist point. As it became increasingly clear that life itself was an information system that could be understood in terms of signs, symbols, and code, and on the basis of the idea that the social, political, economic, and cultural systems could similarly be understood in terms of information transfers and feedback systems, the structuralists began to develop a vision of a system capable of taking in understandings of the natural and human worlds drawn from a range of different academic disciplines, including semiotics, sociology, history, psychoanalysis, literature, and biology. We know that Lacan’s particular contribution to this field was to re-read psychoanalysis through cybernetic and information theory and seek to project the Freudian notion of the unconscious onto the language systems that enables the individual to operate in social context.

In this regard, Lacan [17] starts his reading of *Beyond the Pleasure Principle* [6], Freud’s key work on the ex-tremism of human life, or, in other words, its relationship to an outside, by noting that the key achievement of psychoanalysis was to decentre the modern individual and establish its ex-tremism with regard to its own psychological function. Thus, Lacan says that the Freudian self is always other, whether this refers to the other inside the self (the unconscious blind spot that means that we are never fully transparent to ourselves) or the truly other who similarly remains opaque and more or less unknowable, and develops his reading of the key Freudian machine, the compulsion to repeat, on this basis. Given that we feel constantly decentred, in a state of deficit, and in need of completion, Lacan explains that the human might be understood in terms of a cybernetic feedback machine. In this view we are forever in search of something, some object, or some other to complete ourselves through chains of signification (signs, symbols, objects and so on) that we might boil down to representations that resolve the problem of lack (creating a sense of being), only to later fail (leading to a renewed sense of lack), resulting in the repetition of the entire process over again. In this sense we can see that in Lacan’s Neo-Freudian reading this is a fatal strategy, which never succeeds because the true aim of desire to escape back to the peace of mother is prohibited by the Oedipus complex that ensures entry into language itself, leading to the emergence of an endlessly repetitive machine, a kind of human feedback loop. Essentially, then, this is Lacan’s [17] revision of *Beyond the Pleasure Principle* [6] and the idea of the viral death drive that simultaneously deadens the individual caught in an endless cycle of repetitious pre-post-human behaviour and describes a perfectly functional cybernetic machine cycling backward and forwards between states of nothingness (lack) and being (excess). Here, we see how Freud’s two versions of viral law, on the one hand (a) the fatal death drive and on the other hand (b) the endless life drive, collapse back towards one cybernetic state of feedback, which centrally excludes the possibility of true human life founded upon the idea of freedom beyond mere microbial/mechanical behaviour.

Although there is no true sense of freedom in Lacan’s real, where the human is a body in pieces, his (Lacan’s) first machine, the imaginary mirror relation that alienates the subject from themselves, subordinates humanity to the image. While the subject might prefer to remain inside this basic circuit of the real and imaginary self, Lacan says that the unconscious dream machine ensures that they are constantly reminded of their lack caused by their alienation from nature (mother) and leap into language and symbolic civilization. This is when we (humanity) find ourselves caught up in the repetitious logic of drive and the virus that is precariously balanced on the edge of life and death, being and nothingness, and the relentless pursuit of some other that could make the self complete. As we have seen above, this pursuit of the other, in the form of an endless parade of signs, boils down to the basic mechanism Freud identified in *Beyond the Pleasure Principle* [6]. Everything comes back to fort/da and the attempt to fend off trauma, lack, and displeasure, which Freud then transformed into the inertia of nature, and eventually the death drive back to nothingness that we speculated the founder of psychoanalysis may have developed in order to make some sense of the Spanish flu virus tearing through the population of the world. Following this insight, the next step in my argument was to suggest that Freud recoiled from this desperate conclusion, turning towards Weismann’s germ cell and protozoan to imagine the immortal condition of microbial life that simply divides in order to keep going. Given this backdrop, what the Lacanian [17] move, which we remember entails the collapse of Freud’s two viral laws into one system comprising a cybernetic re-reading of the Freudian self caught in a feedback loop that looks like a kind of living death (the first viral law) *and* a perfectly functional machine able to keep working endlessly into the future (the second viral law), throws up is the precise problem of human freedom somewhere between simple microbial life that lives in order to divide and divides in order to live with no need for otherness, sex, or death and the artificial techno-scientific mechanical simulation of life (automation, the automaton) that works in order to works and keep working and nothing more.

It is precisely this problem of freedom that Derrida [7] addresses in his seminar on Jacob, Freud, and life and death in the context of the American century, the rise of techno-science, cybernetics, and molecular biology. In this work the idea of grammar and general textuality set out in *Of Grammatology* [22] is no longer simply about human culture and language systems opened up by processes of socialisation, but life itself, or, in Jacob’s language, the code of the living, which is endlessly produced and reproduced by the biological program passed down through the generations. In this regard, Jacob’s molecular biology shows how the living is a cybernetic information system founded upon principles of organisation, communication, and control in order to ensure effective functionality. Given this understanding of life, we would have to conclude that an organism might be understood through a process of decoding in much the same way that we might decode the meaning of Joyce’s *Finnegans Wake* [23], which he wrote in order to try to capture the movement towards cybernetic culture, or Schreber’s *Memoirs of My Mental Illness* [24], which many have speculated encoded the Judge’s paranoid schizophrenic efforts to escape from the traumatic reality of past abuse at the hands of the authoritarian father figure who refused to accept difference. Building upon Weismann’s theory of the germ cell that lives on beyond that somatic cell that dies off, which we remember Freud took up in his second theory of viral law, and the discovery of the coding system of DNA in the early 1950s, Jacob explains that the molecular level of the living is programmatic. In this situation Weismann’s germ cell that lives on becomes the bio-cybernetic program that is passed on from generation to generation in the name of reproducing life.

Thus, the program represents a kind of unconscious, deep- or infra-structure that provides the basic instructions for the reproduction of life. Although change is possible through copying errors and selection, Jacob is clear that this system is primarily about repetition and that evolutionary change happens only very gradually through advantage gained in the wake of accidental mutation. Akin to Weismann’s germ cell, Freud’s concept of the death drive, Lacan’s cybernetic switching system, and what we have called viral law, Jacob’s program is not subject to conscious transformation or wilful changes of course. The key point here is that there is no freedom, no other, no outside in Jacob’s molecular biology. According to this thesis, Jacob repeats Weismann’s conclusion, which Freud would in turn repeat in chapter six of *Beyond the Pleasure Principle* [6], that sex and, as a consequence, death came later to the story of evolution when the living took the form of complex, multi-cellular organisms. Following Weismann and Freud, who translated the primitive viral death drive into a modern scientific viral life drive, Jacob concludes that the most basic form of molecular life is eternal and simply divides in order to survive. At this point, and in order to explain the difference between simple and complex life forms, Jacob describes two forms of memory – genetic memory and mental memory. While genetic memory is rigid, unchanging, repetitious, and operates at the level of species, or even beyond this, the living in the widest sense, mental memory is flexible, able to learn, but is also subject to forgetting, death, and finitude. The cost of freedom and the ability to change is, therefore, finitude and death in a wider context defined by a programmatic infrastructure which prohibits wilful change. Commenting on this molecular theory, Derrida notes the connection between Jacob’s law of reproduction and Freud’s Oedipal story, which lifts the idea of programmatic biology towards the level of psycho-social socialisation or, we might say, normalisation. In this case, mother and father produce a child, who is then subject to processes of Oedipal normalisation in order to ensure civilization endures and survives.

Of course, Derrida’s [7] point is that if the basic biological coordinates of the living are programmatic, and organised around a system of communication and control set upon the linear transmission of genetic material across the generations, then it is easy to understand how the Freudian system could be defended on a psycho-social level as somehow natural and part of the evolution of the functional bio-social order. This is Derrida’s target. In my reading of his seminar, he looks to question Jacob’s model of molecular biology in order to throw into question the whole Freudian bio-social system of normalisation premised upon a similar idea of repetition and the reproduction of the same. In his reading of *Beyond the Pleasure Principle* [6], Derrida sees binding and the mastery of trauma as the key idea that Freud never really addresses. In this view the entire purpose of the pleasure principle, the death drive, and in my reading the second version of viral law, is to armour the self and somehow evade the chaos, anxiety, and noise produced by internal excitation and interaction with others in the outside world. As we have seen, this is the essence of Freud’s conservatism concerned with escaping trauma and protecting the ego and the reason he ends up exploring Weismann’s work. Concluding that death is one way out, and that the dead no longer have to manage the pain of loss that seemed to be everywhere when he was writing his masterpiece, we have seen that Freud changes direction and imagines endless life free of otherness and an outside. This is Freud’s utopia that Derrida suggests rests on his own fear of mortality and inability to cope with the enormous scale of loss he witnessed from 1914 onwards. While this retreat towards the immortality of microbial life is no doubt understandable, I think that Derrida’s [7] basic problem with Freud’s move is that it really undercuts the wider purpose of psychoanalysis which is to reflect, understand, make sense, and then on the basis of this knowledge to exercise human freedom.

It would seem to me that this is precisely what Freud [6] trades away in the name of thinking through a deep structure of immortal life that could ease the pain of his state of unmourning and Lacan [17] would similarly abandon in his theory of the cybernetic technological self taken up in his own reading of *Beyond the Pleasure Principle*. In identifying the same kind of deep structure operating in Jacob’s [18] molecular biology and a cybernetic reading of the function of DNA, Derrida’s identifies a kind of hidden Hegelianism operating within the new science of the living. Although we learn that the program has no will, its commitment to the reproduction of a particular type of structure suggests some kind of teleology or teleonomy. Despite the shift from the early modern idea of tele-ology, where progress towards the end (tele) is through reason (logos), to the more techno-scientific concept of teleonomy, where the same progressive change (tele) is through the application of programmatic law (nomos), the point remains that evolutionary change seems to be moving towards some end (telos) of biological development or at least this is what Derrida suspects. If this is, in fact, the case the next question concerns exactly what mechanisms allow this progressive change to take place. It is in thinking through Jacob’s response to this point that Derrida begins to deconstruct the idea of viral law, which we have set out across readings of Freud and Lacan, because it is here that the molecular biologist reluctantly admits the necessity of otherness. According to this thesis, the process that allows the primitive unicellular organism to develop, and, as a consequence the hinge that connects simple to complex life and allows evolution to happen, is what Jacob and then Derrida call viral sex, which describes the process whereby a virus breaches the cell wall of a microbial organism and eventually transform its DNA.

While this process looks like a hostile take-over from the point of view of the unicellular utopia of the same committed to programmatic reproduction without difference, Derrida’s point is that what Jacob admits through his recognition of molecular sex is the fact that difference, otherness, sexuality, and as a consequence the metabolic process of death, exist on the most basic level of the living. In other words, there is no sense in which primordial, molecular life is self-identical, programmatic and possessed by the drive to the endless reproduction of the same, whereas later, more evolved complex life is subject to laws of otherness, relationality, sexuality, and finitude. On the contrary, what Derrida [7] takes from Jacob’s [18] text is the admission that difference is essential, that there is no absolute immunity to the outside, and that the viral law of Weismann, taken up by Freud, and then later Lacan who transformed the idea of endless repetition into a cybernetic mechanism, must be rethought. Given that the programmatic vision of Weismann, Freud, Lacan, and, in the case of molecular biology, Jacob appears to represent a kind of idealistic utopia model of once and for all immunity, Derrida’s version of viral law becomes absolutely about understanding the virus as a kind of primordial agent of change and driver of evolutionary transformation. Against the microbial, molecular conservatism of Weismann, Freud, and Jacob, Derrida comes to understand the figure of the virus as a microscopic revolutionary, capable of destabilising self-identical structures in the name of difference, change, and potential futures beyond the simple reproduction of the same.

## Viral Futures

In Derrida’s [7] reading the virus is no longer Freud’s spectral death drive that rips through the population, leading him to imagine a very different kind of viral law of the endless reproduction of life, or Lacan’s relentless high-tech switching system that catches the cybernetic self within a kind of eternal feedback loop that looks like a perfect machine *and* a funereal vicious circle comprising a monstrous confusion of life and death. Instead, Derrida’s virus is the primordial supplement that introduces sex, death, and the possibility of evolutionary transformation into what would otherwise look like a completely closed system of the living unable to develop and change. In this respect we might conclude that the idea of the virus, and the notion of viral law, speaks to Derrida’s concept of the pharmakon, which is simultaneously a cure and a poison. In this interpretation, which recalls Carl Zimmer’s [25] point about the etymology of the Latin word ‘virus’, referring to both seminal fluid and poisonous toxin, the meaning of viral law encompasses the various perspectives we have set out above: the Freudian story of the relentless death drive and the unstoppable life force of the germ cell; the Lacanian re-reading of Freud where drive becomes perfect mechanisation that looks a lot like living death; and finally Derrida’s story of evolution through the emergence of difference, sex, and death. We know that Derrida’s problem with the logic of repetition and sameness set out by Freud and Lacan is that he thinks that what this is really about is a will to mastery and control that smacks of authoritarianism. In this theory Freud’s endless struggle between on the one hand, the pleasure principle that pushes the organism towards suicide in the name of escape from the traumas of life and on the other hand, the reality principle that demands that the self holds back, exercises restraint, and endures the horror of impending death, masks what all of this is really about – psychological control and mastery over a threatening world. This is, of course, precisely what we can see in Freud’s own biography living through the horror of World War I, the Spanish flu pandemic, and beyond into the 1930s. We have speculated that he wrote *Beyond the Pleasure Principle* [6] in order to masters his own feelings relating to the pervasive atmosphere of death that fell over Europe in the early 20th century and centrally in order to make sense of the loss of his own daughter Sophie, mother of Ernst who played fort/da to cope with her disappearance.

We have seen that this situation may have led Freud to mythologise the flu virus in the idea of the death drive, only to then take off in an alternative direction in order to suggest a utopian story of unicellular immortality and unstoppable life that knows no other. Following Lacan’s re-reading of the death drive in terms of the idea of a perfect cybernetic system that seems to destroy the possibility of life in a kind of terminal feedback loop, we took up Derrida’s reading of Jacob’s molecular biology, which takes in Freud’s idea of microbial immortality and builds upon Lacan’s insight that the perfectly functioning machine ends up creating a state of living death. As a result, Derrida critiques Jacob’s molecular theory of programmatic life as a fable of the possibility of endless generation, growth, and development without the limit of otherness or sexuality, which necessarily introduces the brake of finitude and death. It is in responding to this bio-cybernetic fantasy, which, if we were to evoke Heidegger [26] we might connect to the development of America in the second half of the 20th century, that Dawne McCance [27] explains the importance of Derrida’s intervention, explaining that the techno-scientific fantasy of the reproduction of the same is precisely what has led humanity into the black hole of the Anthropocene that we seem unable to think beyond precisely because we cannot think in terms of otherness. Perhaps this is what the virus really represents? Perhaps this is how we must understand the cultural politics of the virus? Concerning the fantasy of the endless viral reproduction of the same that we find in Weisman, Freud, Lacan, and Jacob, McCance makes the argument that we need to challenge the sovereignty of the molecular biological paradigm precisely because of the ways in which it imposes a vision of a kind of nihilistic drive upon the ways in which we think about the living, but also the human social, political, economic, and cultural system, which, in this view, is entirely unable to change. We know that the neoliberals think that there is no alternative, but what molecular biology does is to ground this principle of unfreedom in the very molecules of life itself. If this is how we have ended up staring down the barrel of Anthropocentric extinction, then we must think clearly about the significance of the virus. Beyond its terrible destructive impact upon the lives of so many individuals, the Covid-19 virus might be seen to challenge the sovereignty of the biological project concerned with the reproduction of sameness and suggest that change can happen at a social, political, economic, and cultural level. In this regard we must recognise that it is not only the mortal body of the individual that perishes, which is, in a sense something that we have always known, but also the sovereign, apparently immortal body of the species and the living that projects itself into the human socio-politico-economic and cultural systems which will not endure unless we understand the need to embrace otherness and change our ways. Given the trauma of the past two years, perhaps this is how we should understand and make sense of the cultural politics of Covid-19 and what I am calling viral law.
